# Mind the Missing Gap: A Cervical Variant of Type A Esophageal Atresia

**DOI:** 10.3390/children12060740

**Published:** 2025-06-06

**Authors:** Marco Di Mitri, Riccardo Coletta, Edoardo Collautti, Cristian Bisanti, Annalisa Di Carmine, Roberto Lo Piccolo, Elena Rovero, Francesca Tocchioni, Elisa Severi, Marco Moroni, Ioannis Georgopoulos, Dariusz Patkowski, Mario Lima

**Affiliations:** 1 Pediatric Surgery Department, Meyer Children’s Hospital IRCCS, 50139 Firenze, Italy; riccardo.coletta@meyer.it (R.C.); edoardo.collautti@studio.unibo.it (E.C.); roberto.lopiccolo@meyer.it (R.L.P.); elena.rovero@unifi.it (E.R.); francesca.tocchioni@meyer.it (F.T.); elisa.severi@meyer.it (E.S.); 2 Alma Mater Studiorum, University of Bologna, 40126 Bologna, Italy; 3 Department of Neurofarba, University of Florence, 50121 Firenze, Italy; 4 Pediatric Surgery Department, IRCCS Sant’Orsola, Alma Mater Studiorum, University of Bologna, 40126 Bologna, Italy; cristian.bisanti@studio.unibo.it (C.B.); annalisa.dicarmine@studio.unibo.it (A.D.C.); mario.lima@unibo.it (M.L.); 5 Neonatal Intensive Care Unit, Meyer Children’s Hospital IRCCS, 50139 Firenze, Italy; marco.moroni@meyer.it; 6 Department of Surgery, Aghia Sophia Children’s Hospital, 115 27 Athens, Greece; 7 Department of Pediatric Surgery and Urology, Wroclaw Medical University and Hospital, Borowska 213, 50-556 Wroclaw, Poland; dariusz.patkowski@umw.edu.pl

**Keywords:** esophageal atresia, type A esophageal atresia, cervical esophageal atresia, primary anastomosis, long gap esophageal atresia, thoracoscopic surgery, minimally invasive surgery

## Abstract

**Background:** Esophageal atresia (EA) type A, characterized by the absence of a tracheoesophageal fistula and typically presenting with a long esophageal gap, usually requires staged repair. **Methods:** We report a rare case of a newborn with type A EA in which both the proximal and distal esophageal pouches were unexpectedly close and located in the cervical region. This anatomical variant allowed for a successful primary anastomosis through a cervical approach. **Results:** Initial imaging was misleading, and the true anatomy was clarified only through thoracoscopic exploration, underscoring the importance of intraoperative flexibility. **Conclusions:** To our knowledge, this is the first report of such a presentation in type A EA, with significant implications for diagnosis and surgical strategy.

## 1. Introduction

Esophageal atresia (EA) is a rare congenital anomaly resulting from incomplete esophageal development during foregut embryogenesis. It has an estimated incidence of 1 in 3500 live births and is commonly associated with one or both pouches connecting to the trachea, a condition called tracheoesophageal fistula (TEF). Embryologically, the esophagus develops from the foregut, and disturbances in longitudinal growth or foregut separation may result in a spectrum of anatomical configurations [1]. The most widely used classification system, proposed by Gross, describes five anatomical types (Table 1) [2].

Among these, type A (7–8% of cases) is characterized by two esophageal pouches typically widely separated, with the proximal pouch ending in the upper thorax and the distal pouch in the lower mediastinum. This substantial gap often required staged surgical approaches, including thoracoscopy, gastrostomy, delayed primary anastomosis, esophageal replacement techniques, or esophageal lengthening procedures such as the Foker process [3,4,5].

Furthermore, accurate preoperative identification of the esophageal pouches remains challenging, especially in neonates. While radiographic findings—such as absence of intra-abdominal gas—strongly suggest type A EA, they offer limited insight into pouch length or location. This often necessitates intraoperative exploration to determine the most appropriate surgical approach.

We present a unique case of type A EA in which both esophageal pouches were ultimately found to be in the cervical region, an unexpected finding revealed during thoracoscopic exploration, which initially showed the distal pouch extending cranially to the thoracic inlet. The patient, after thoracoscopic exploration underwent to a successful primary anastomosis through a cervical approach. To our knowledge, this is the first reported case of type A EA with both esophageal pouches located in the neck, representing a rare anatomical variant with implications for diagnosis and surgical management.

## 2. Case Report

A male 36 + 3/40 gestational age newborn, birth weight 3136 g, was naturally delivered in a district general hospital due to severe polyhydramnios (AFI: 35 cm). Prenatal ultrasound had shown severe polyhydramnios without evidence of gastric bubble, raising suspicion for esophageal atresia. No other congenital anomalies were detected during prenatal screening. A preoperative bronchoscopy was performed prior to the gastrostomy in order to rule out a proximal tracheoesophageal fistula. The procedure confirmed the absence of a fistula and revealed no signs of tracheomalacia.

**Figure 1:**
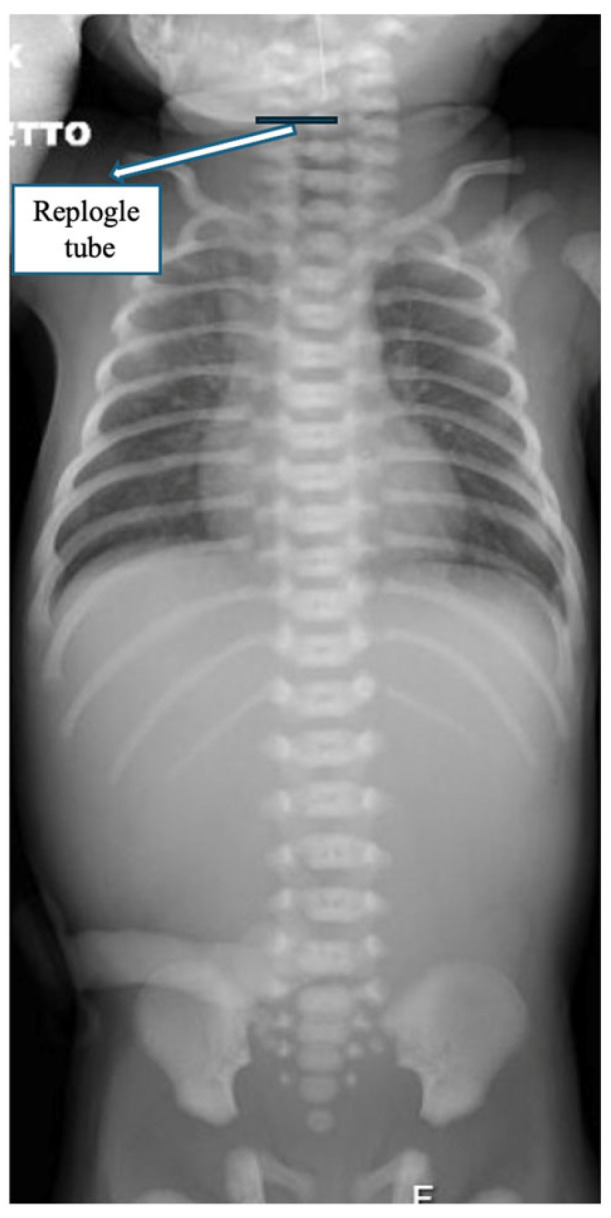
The esophageal gap and the X-ray showing the replogle tube in the neck and the absence of intra-abdominal gas.

At birth, the baby presented with excessive salivation. Attempts to pass a nasogastric (NG) tube were unsuccessful beyond 9 cm at the lips, indicating an obstruction. The tube was left in place at that level, and a babygram obtained with gentle downward pressure showed the tube coiled in the upper mediastinum and absence of intra-abdominal gas, consistent with esophageal atresia type A (Figure 1).

Based on initial radiographic findings suggestive of a long-gap EA, a gastrostomy was performed on the first day of life to ensure enteral nutrition while planning for delayed primary repair. The patient was scheduled for thoracoscopic assessment after 15 days to evaluate the esophageal gap and determine the need for traction techniques or other staged approaches. However, intraoperative findings revealed that the esophageal segments were in close proximity and both located in the cervical region, allowing for immediate primary anastomosis via a cervical approach.

Due to the radiological findings, a conventional pathway for this patient was enrolled; on the first day of life, the patient underwent gastrostomy placement to allow enteral feeding while awaiting delayed primary anastomosis. The x-ray study performed through gastrostomy showed very shorth distal pouch ending at the level nearly above the diaphragm.

**Figure 2:**
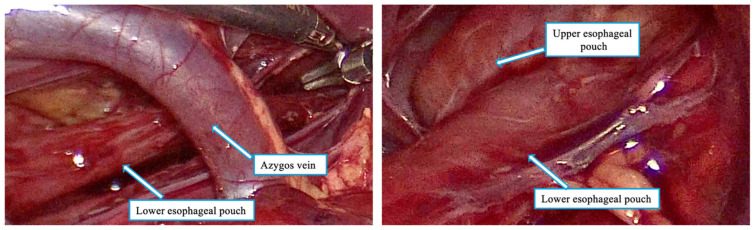
Intra-operative findings: the lower esophageal pouch extending under the azygos vein ending very close to the upper esophageal pouch at cervical level.

After 15 days, the patient was taken to the operating room for thoracoscopic exploration. Unexpectedly, a regular distal esophageal pouch was visualized, extending cranially up above the thoracic inlet and appearing to reach the cervical region (Figure 2).

**Figure 3:**
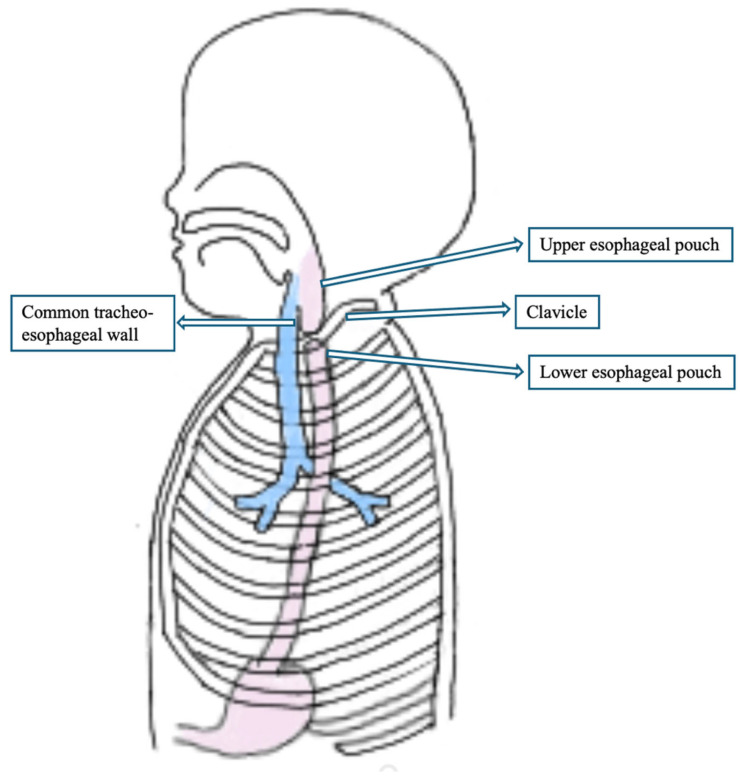
Anatomical point of view: both esophageal pouches located in the cervical region, allowing primary anastomosis via a cervical approach.

The thoracoscopic assessment was performed with the aim of measuring the esophageal gap and evaluating the feasibility of primary anastomosis or internal traction with delayed primary anastomosis. However, intraoperative visualization of the distal esophagus extending into the thoracic inlet prompted reconsideration of the surgical plan (Figure 3).

Therefore, a stay suture was placed at the most cranial portion of the distal esophageal stump, which was identified at the clavicular level, entirely outside the thoracic district. Although thoracoscopy revealed the proximity of the two esophageal ends, the anastomotic site was located extremely cranially, near the thoracic inlet. The confined space at that level made it technically unfeasible to safely place sutures via thoracoscopy. Therefore, a cervical approach was preferred to ensure optimal exposure and minimize the risk of complications.

**Figure 4:**
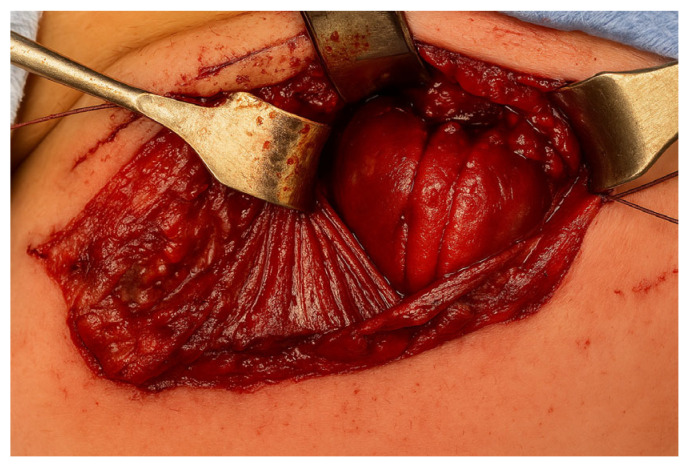
The end-to-end esophageal anastomosis by cervical approach.

A right-sided cervicotomy was performed, through which both the proximal and distal esophageal pouches were identified very close together. Therefore, a primary end-to-end anastomosis was successfully performed without tension using interrupted absorbable sutures (Figure 4).

**Figure 5:**
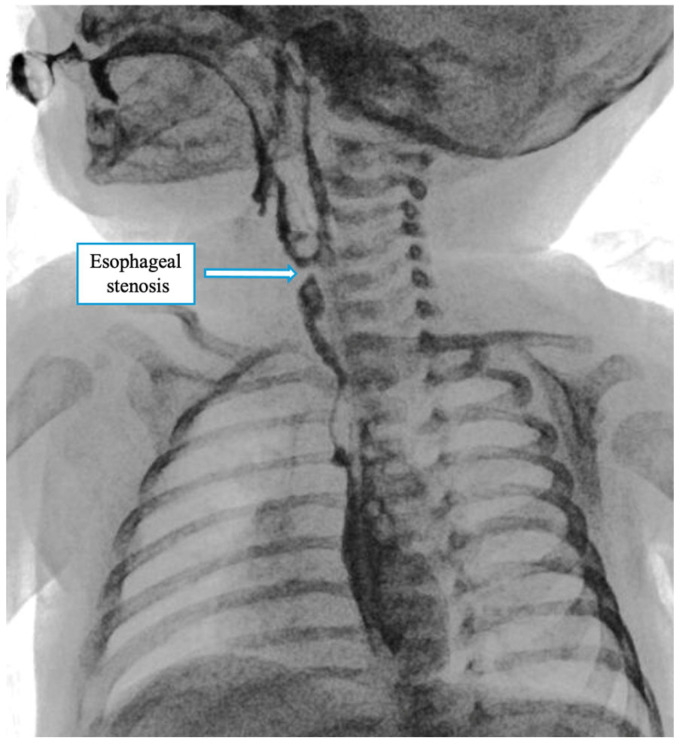
Postoperative contrast study on day 7 showing a mild narrowing at the anastomotic site, consistent with a benign stricture. No contrast leakage was observed.

A contrast X-ray performed on postoperative day VII showed stenosis at the anastomotic site (Figure 5). The patient underwent serial esophageal dilations (10 session) up to a caliber of 10 mm, and until clinical stability and good tolerability of oral feeding were achieved. During this period, oral intake improved gradually, and by the end of the third month, the patient had achieved stable, full oral feeding. At the latest follow-up at 6 months of age, the child was tolerating an age-appropriate oral diet without feeding-related difficulties, and growth parameters were within the normal range.

## 3. Discussion

Esophageal atresia (EA) type A, characterized by the absence of a tracheoesophageal fistula, presents significant surgical challenges due to the typically wide gap between the proximal and distal esophageal segments. In most cases, the upper pouch ends in the upper thoracic cavity. At the same time, the distal segment lies deep in the posterior mediastinum, making primary anastomosis technically difficult. The case presented deviates significantly from this classic anatomical pattern, with both esophageal segments located in the cervical region and in proximity, permitting a primary repair through a cervicotomy. This represents the first documented instance of such an anatomical variant in Gross type A EA.

Kemmotsu et al. presented the cervical approach for cases of EA with TEF (type C) located in a high position emphasizing the role of bronchoscopy or contrasty study to detect the location of the distal pouch [6,7]. However it would be not helpful in our case as there was no fistula so eventual bronchoscopy would bring nothing to the diagnosis.

Also, Nakagawa et al. presented two case with EA type C approach by the neck, suggesting to perform the preoperative contrast examinations and bronchoscopy routinely to detect the location of the upper esophageal pouch and of the TEF [8]. However, contrast studies are not routinely performed in type C EA. The diagnosis is usually made based on clinical signs and standard radiography. Bronchoscopy remains the key preoperative tool to locate the fistula and evaluate the anatomy. Contrast studies may be useful in selected cases, particularly when a high or cervical fistula is suspected or the anatomy is unclear.

Contrast study is suitable when EA associated with TEF while in our case it’s not possible estimate the length of the distal pouch because of the absence of the fistula. Although the contrast study through the gastrostomy suggested a very short distal pouch, this was misleading. The distal esophagus was extending cranially into the cervical region, as revealed during thoracoscopic exploration. This highlights the limitations of contrast studies in type A EA without fistula, where the true anatomy may not be accurately represented.

This unusual presentation may have important implications for both diagnosis and surgical strategy. In standard practice, preoperative imaging can provide valuable information about pouch location, although it may be limited in precisely identifying the extent of the distal esophagus, particularly in neonates. Despite typical radiological findings suggesting long-gap atresia, the proper anatomy only became evident during thoracoscopic exploration. The cranial course of the distal esophagus, extending into the cervical region, is highly atypical and not previously described in the literature. Identifying a typical distal pouch with cranial extension supports the hypothesis of a developmental arrest at a higher anatomical level than usual, possibly due to an aberration in the longitudinal growth or separation of the foregut during embryogenesis [9]. Our case showed clear the advantage of thoracoscopic approach that was very helpful in correct diagnosis in atypical situation.

This rare anatomical variant highlights the need for intraoperative adaptability. While thoracoscopy remains the preferred diagnostic approach in such cases—thanks to its minimal invasiveness and superior visualization—esophageal continuity can also be assessed through an open procedure when necessary. In our case, thoracoscopy could not be continued due to the exceptionally high cervical location of the esophageal ends, which limited the operative space and prevented precise needle manipulation. Therefore, the procedure was converted to a cervical approach. Cervical anastomosis is generally associated with lower morbidity and facilitates easier postoperative management, including dilations. In our patient, the postoperative course was uneventful, with successful recovery of oral feeding following only a few sessions of esophageal dilation to address a mild anastomotic stricture. From a broader perspective, this case highlights the heterogeneity of EA anatomy and suggests that not all Gross type A cases should be assumed to have a long-gap configuration. In addition to its surgical relevance, this case may serve as a useful contribution to basic anatomical and developmental research. The presence of both esophageal pouches in the cervical region suggests a possible embryological arrest or deviation at a higher level than typically observed in type A EA. This observation supports further investigation into the developmental mechanisms underlying esophageal elongation and separation. From a diagnostic standpoint, the case also raises important considerations: in the absence of a tracheoesophageal fistula, the distal esophageal pouch is often not easily visualized, limiting the utility of preoperative contrast studies via gastrostomy. Although such studies might be attempted to estimate pouch length, their accuracy is questionable without a patent fistula. In these situations, thoracoscopic exploration remains the most reliable tool to assess anatomy and guide the surgical strategy.

## 4. Conclusions

This case describes a rare anatomical variant of Gross type A esophageal atresia, with both esophageal pouches in the cervical region, allowing for primary anastomosis via a cervical approach. While the cervical approach has been rarely described for esophageal atresia with tracheoesophageal fistula, its application in type A EA is unprecedented. It shows the advantage of thoracoscopy to precisely check the malformation anatomy. If thoracoscopy was performed as the first procedure it even would be possible to avoid gastrostomy and shortage the time of hospital stay. Improved preoperative assessment and early thoracoscopic exploration may help identify such rare anatomical variations, optimizing surgical outcomes.

## Figures and Tables

**Table 1:** Gross’s classification of esophageal atresia.

Gross Classification of EA	
Type A	EA without TEF
Type B	EA with proximal TEF
Type C	EA with distal TEF
Type D	EA with both proximal and distal TEFs
Type E	Isolated TEF without EA

## Data Availability

No new data were created or analyzed in this study. Data sharing is not applicable to this article due to the nature of the case report.
